# Effect of 9% Hydrofluoric Acid Gel Hot-Etching Surface Treatment on Shear Bond Strength of Resin Cements to Zirconia Ceramics

**DOI:** 10.3390/medicina58101469

**Published:** 2022-10-17

**Authors:** So-Hyun Kim, Sung-Chan Cho, Myung-Hyun Lee, Hyo-Jung Kim, Nam-Sik Oh

**Affiliations:** 1 Department of Dentistry, Inha University School of Medicine, Inha University Hospital, Incheon 22332, Korea; 2 Department and Research Institute of Dental Biomaterial and Bioengineering, Yonsei University College of Dentistry, Seoul 03722, Korea; 3 Energy & Environmental Division, Korea Institute of Ceramic Engineering and Technology, Jinju 52851, Korea; 4 Department of Dentistry, University of Ulsan College of Medicine, Ulsan University Hospital, Ulsan 44033, Korea

**Keywords:** hydrofluoric acid etching, resin cement, shear bond strength, zirconia ceramics, zirconia surface treatment

## Abstract

*Background and Objectives*: There is no consensus regarding the surface treatment method for achieving optimal bonding strength between zirconia and resin cements. We evaluated the effect of hot-etching with 9% hydrofluoric acid (HF) gel using the Zirconia Etchant Cloud System on zirconia surfaces and the consequent shear bond strength (SBS) of different resin cements to such surface-treated zirconia ceramics. *Materials and Methods*: Forty-five zirconia specimens were randomly assigned to surface-treatment groups (n = 15/group): no treatment (control, CT); sandblasting with 110-μm Al_2_O_3_ at an air pressure of 1 bar for 10 s (SB); hot-etching with 9% HF gel (HE). Post-treatment, specimens were examined using scanning electron microscopy (SEM) and surface roughness (SR) analysis. After treatment, self-adhesive resin cements (Maxcem Elite, MAZIC Cem, RelyX U200, 3M ESPE: Maplewood, MN, USA) were bonded to zirconia specimens, which were stored in distilled water at 37 °C for 24 h. All specimens were then subjected to SBS testing, using a universal testing machine, until failure. Data were analyzed using one-way analysis of variance and Tukey’s post hoc test (α = 0.05). *Results*: In the SEM images, roughness was greater in SB than in HE specimens. Ra and Rt values were highest in SB, followed by HE, and CT specimens. HE specimens showed significantly higher SBS values than CT or SB specimens (*p* < 0.05). MAZIC Cem cement, with 10-methacryloyloxydcyl dihydrogen phosphate yielded the highest SBS values. *Conclusions:* Hot-etching with 9% HF gel in a safe shell formed uniformly small, defined holes on the zirconia surface and achieved significantly higher SBS values than sandblasting (*p* < 0.05). Zirconia prostheses can be bonded micromechanically with resin cement, without the deterioration of properties due to t-m transformation, using chemical acid etching with the Zirconia Etchant Cloud System.

## 1. Introduction

Due to high gold prices, and aided by advances in dental computer-aided design and computer-aided manufacturing (CAD/CAM) systems, the clinical use of zirconia in dentistry has increased exponentially, with zirconia ceramics now rapidly replacing gold and metal restorations [1,2]. Dental zirconia is supplied mainly in block form, as yttria-stabilized pre-sintered zirconia, and is then milled using dental CAD/CAM techniques to obtain a final prosthesis. Zirconia has superior mechanical properties, such as high flexural strength, fracture resistance, tooth-like esthetic appearance, high chemical stability, and biocompatibility [1,2,3,4] High-strength all-ceramic prostheses, including those made of zirconia, can be cemented using zinc phosphate or modified glass-ionomer cement. However, resin-based luting cements are the best adhesive material in terms of margin seal, retention, and fracture resistance [4,5]. Nevertheless, compared to glass-ceramic materials, zirconia prostheses exhibit poor adhesion to resin cement [4,6]. Strong adhesion to resin cement can improve retention, prevent microleakage, and increase the fracture/fatigue resistance of zirconia prostheses [2].

Various mechanical and chemical surface treatment methods have been applied to increase the adhesive area and enhance the bond strength of zirconia ceramic and resin cement [7,8,9,10,11]. Sandblasting is the most widely used method for mechanical surface treatment intended to provide roughness to ceramic surfaces. However, sandblasting is affected by various variables, such as abrasive grain size and spraying distance [12]. In particular, when close-sprayed with large grains, the mechanical properties of zirconia prostheses deteriorate, leading to formation of tiny cracks on the surface because the zirconia phase transitions from tetragonal to monoclinic (t-m transformation) due to the strong thermal shock. Such change to the monoclinic phase has a marked impact on the long-term stability and fracture of the zirconia restorations [2,13,14]. Blatz et al. recommended sandblasting of zirconia surfaces followed by use of a resin cement containing a 10-methacryloyloxydcyl dihydrogen phosphate (MDP) monomer [15]. This resin cement is used because the phosphate ester monomer of 10-MDP chemically bonds with the hydroxyl groups of zirconia ceramics [16,17,18,19,20].

Several other methods for achieving roughness of the zirconia surface have been recommended [21,22,23]. Among them, the acid etching method chemically corrodes the zirconia surface and increases its roughness [24]. The chemical etching methods have the advantage that it can produce surface roughness without significantly affecting the properties of the zirconia [25,26]. It has been confirmed that a high-concentration hydrofluoric acid solution mixed with various strong acids increases the roughness of the zirconia surface [8,27]. Zirconia is not etched by HF at room temperature because it is a non-silica-based ceramic [2]. However, high-temperature immersion in a high-concentration HF solution for a long period of time makes it possible to etch a zirconia surface [25,26,28]. According to Casucci et al., zirconia treated by hot-etching with a low concentration of acid had a roughness similar to that of zirconia treated with a high concentration of acid at room temperature [21]. However, the hazards of HF are well recognized. When working with HF, an isolated workspace with adequate ventilation is necessary. HF is not a particularly strong acid, but it diffuses into cells and kills them by disrupting their metabolism. When HF is spilled onto the skin, no immediate burn may appear, as it often takes 24–48 h to observe the effects, which can include very deep tissue necrosis. Even a 2% aqueous HF solution can cause corneal erosion [29].

On the other hand, HF gel does not flow easily and can be applied to a small area; therefore, it is relatively safer than the HF solution. This study therefore aimed to evaluate the effect of hot-etching with 9% hydrofluoric acid (HF) gel on zirconia surfaces and the consequent shear bond strength (SBS) of different resin cements to such surface-treated zirconia ceramics. Hot-etching was performed with a 9% HF gel in a safe shell with a triple-locking structure that prevented exposure to hazardous substances, such as the HF vapor. The null hypothesis was that hot-etching with 9% HF gel using the Zirconia Etchant Cloud System would have no effect on the zirconia surface and, hence, would exhibit no increase in the bonding strengths of the various resin cements.

## 2. Material and Methods

Forty-five zirconia specimens (Alpha Z, Dmax Co., Daegu, Korea) were prepared in a rectangular block form with dimensions of 32 × 15 × 5 mm by InLab MC X5 (Dentsply Sirona, Hanau, Germany), sintered according to the manufacturer’s guidelines, and then polished with 1000 grit silicon carbide paper. A Zirconia Etchant Cloud System (Medifive Co., Ltd., Incheon, Korea) containing 9% HF gel was used, which provided a triple-locking safe shell and HF neutralizer, and a heat-generating pack. We used 110-μm Al_2_O_3_ (Eazymill, Vericom Co., Ltd., Chuncheon, Korea) particles for sandblasting. The self-adhesive resin cements used in this study are listed in Table 1.

**Figure 1:**
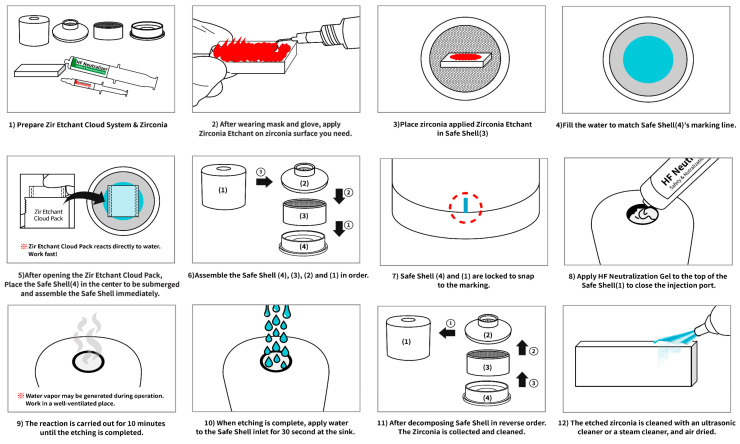
HF hot-etching procedure of the Zirconia Etchant Cloud System.

Forty-five zirconia specimens were randomly assigned to three groups (n = 15/group), according to the surface treatment method used:

1. Control (CT): No surface treatment
2. Sandblasting (SB): Specimens were sandblasted with 110-μm Al_2_O_3_ at an air pressure of 1 bar for 10 s.
3. Hot-etching with 9% HF gel (HE): Specimens were etched using the Zirconia Etchant Cloud System in a safety shell according to the manufacturer’s recommended procedure for 10 min. The manufacturer’s recommended procedure is illustrated in Figure 1.

**Figure 2:**
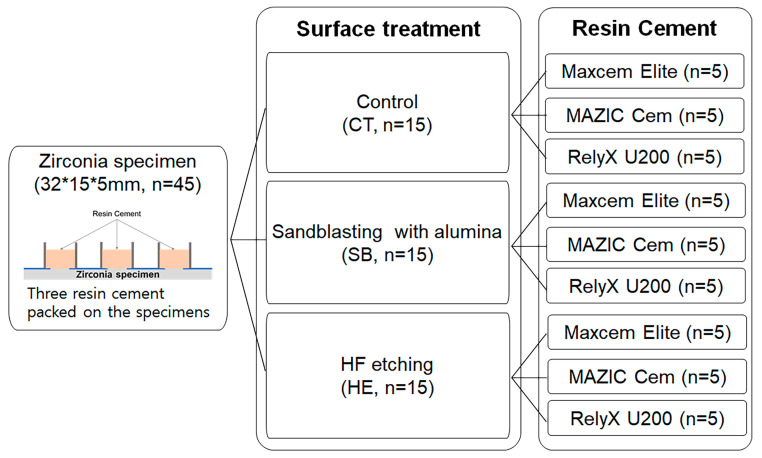
Schematic diagram of experimental grouping.

Grouping of specimens is schematically shown in Figure 2.

**Figure 3:**
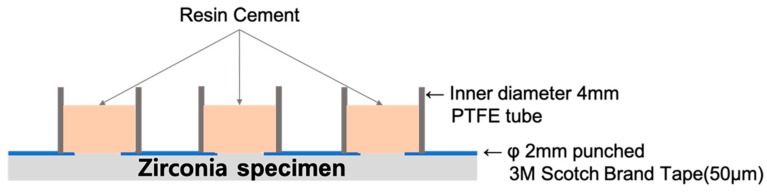
Schematic illustration of making the shear bond strength specimen.

A uniformly sized cement adhesive area was obtained by attaching a 3M Scotch brand tape to a zirconia specimen. The tape had a thickness of approximately 50 μm, with three holes of 2 mm in diameter spaced at regular intervals. Then, polytetrafluoroethylene (PTFE) tubes (internal diameter: 4 mm; external diameter: 6 mm; height: 5 mm) were placed on the adhesive area and filled with resin cement to create cement cylinders 4 mm in diameter and 5 mm in height (n = 15). All resin cement cylinders were self-cured according to the manufacturer’s guidelines, and the PTFE tube was removed after self-curing was completed. Specimens were stored in distilled water at 37 °C for 24 h. The fabrication method of the SBS specimens is shown in Figure 3.

The SBS was measured by applying a vertical force of 2000 N at a rate of 0.8 mm/min to the specimens prepared as described above, using a universal testing machine (Oriental Testing Machine Co., Ltd., Busan, Korea), until failure occurred.

All specimens were mounted on coded brass stubs, sputter-coated with gold for 180 s at 40 mA and examined using a scanning electron microscope (FE-SEM; JSM-6700F, JEOL Ltd., Akishima, Japan), operated at 20 kV.

The zirconia surface was analyzed by X-ray diffraction (XRD) (DMAX 2500, RIGAKU, Japan). The surface roughness (Ra, µm) and peak-to-valley roughness (Rt, µm) were measured using a surface roughness measuring instrument (SE3500, Kosaka Laboratory Ltd., Tokyo, Japan).

All statistical analyses were performed using the IBM SPSS version 24 software (IBM Corp., Armonk, NY, USA). To determine the effects of surface treatment on SBS, the data were analyzed using one-way analysis of variance (ANOVA), with the Tukey post hoc test. Statistical significance was set at *p* < 0.05.

## 3. Results

**Figure 4:**
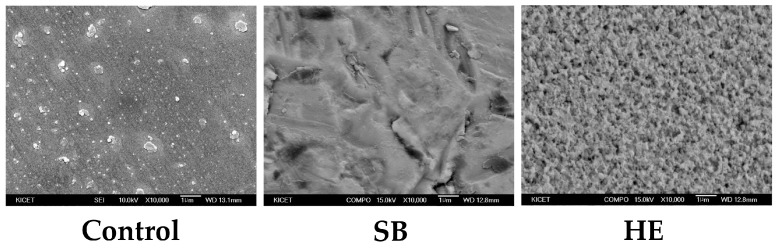
Scanning electron microscopy images of treated zirconia surfaces (Original magnification ×10,000). SB, sandblasted; HE, hot-etched.

The SEM images of the test groups after surface treatment are shown in Figure 4. In the CT specimens, a small amount of debris was observed on the flat surface. In SB specimens, the surface was sharply curved. In HE specimens, small, defined holes were uniformly observed. Although roughness was greater in SB than in HE specimens, micro- and nanoscale porosities were observed on the entire surface of HE specimens.

The surface roughness values of the specimens were also measured. Values of Ra and Rt were highest in SB specimens, followed by HE and then CT specimens (Table 2). SB showed a higher Ra value than HE (*p* < 0.05). Rt exhibited a tendency similar to that of Ra.

**Figure 5:**
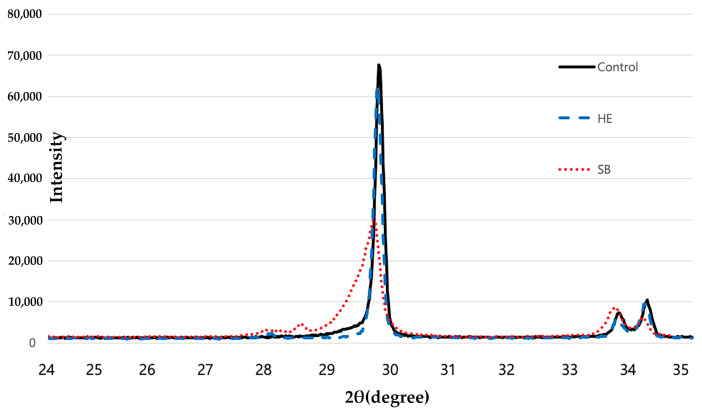
X-ray diffraction patterns of control (CT), sandblasting (SB), and hot-etching (HE) specimens.

The structures of the experimental zirconia specimens were also assessed (Figure 5). Unlike CT and HE specimens, the tetragonal phase was significantly reduced at 30 in SB specimens. The monoclinic phase appeared between 28 and 29, and the monoclinic phase peaks were wide at approximately 29–30. The graph of the SB specimens differed from those of the CT and HE in sections 28–29, 29–30, and 34–35.

**Figure 6:**
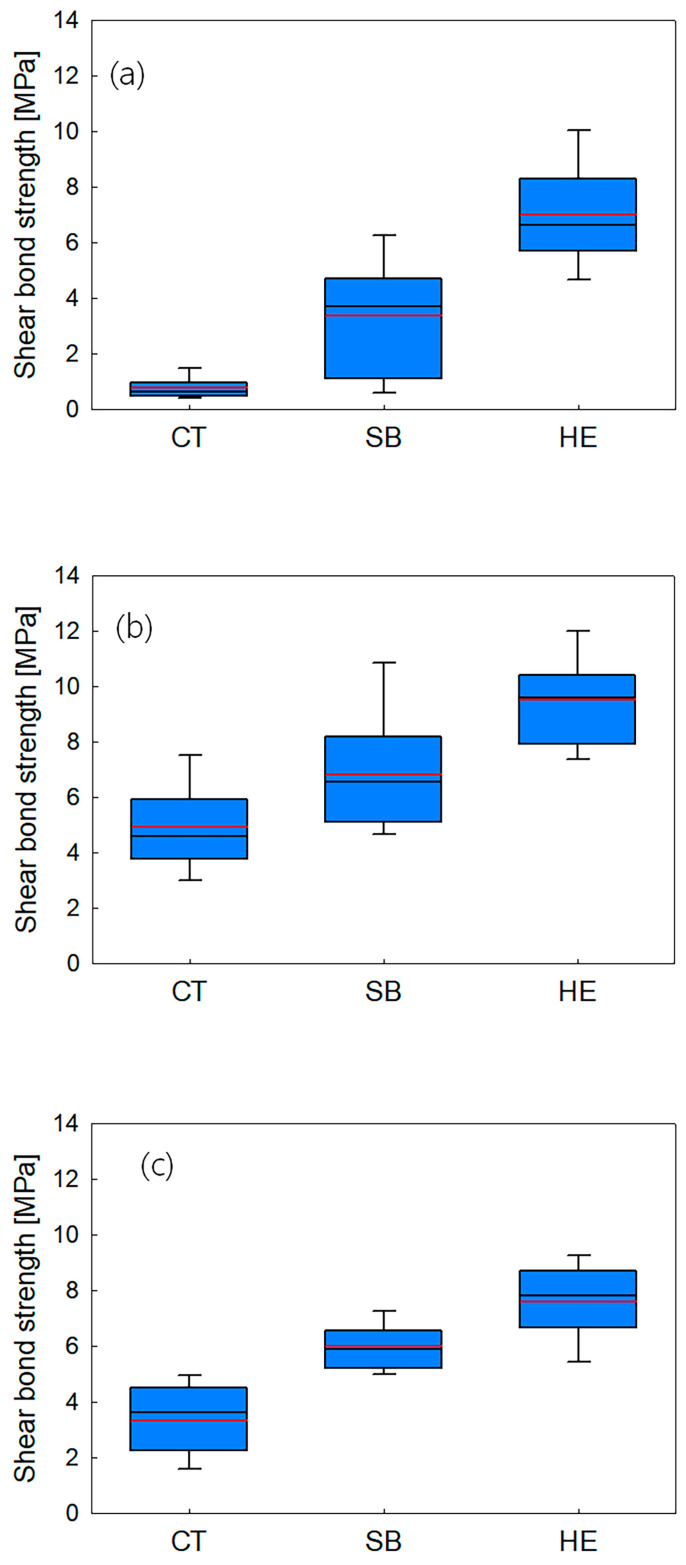
Shear bond strength graph between surface treatments and resin cements: (**a**) Maxcem Elite, (**b**) MAZIC cem, (**c**) RelyX U200. CT, control; SB, sandblasting; HE, hot-etching (*p* < 0.05).

The SBS values achieved with the three types of resin cements (Maxcem Elite, MAZIC Cem, and RelyX U200) were higher in the HE and SB groups than in the CT group (Figure 6, Table 3).

One-way ANOVA revealed a significant difference in the SBS of resin cements to zirconia specimens according to the surface treatment methods (*p* < 0.05) (Table 4). HE specimens showed significantly higher SBS values than did CT and SB specimens (*p* < 0.05). After Tukey post hoc test by rank, HE specimens showed significantly higher SBS values than CT and SB specimens (*p* < 0.05).

## 4. Discussion

In this study, we evaluated the effect of hot-etching with 9% HF gel on the surface roughness of zirconia and on the SBS of self-adhesive resin cements (Maxcem Elite, MAZIC Cem, RelyX U200) to these surface-treated zirconia ceramics. In SEM images, SB specimens showed an irregular surface that was sharply caved, while HE showed small, sharply defined, uniform holes. Surface roughness (Ra) and peak-to-valley roughness (Rt) values were highest for SB, followed by HE, and CT specimens, in descending order. XRD analysis showed that there was a transformation to the monoclinic phase in the SB specimens, as described in other studies [13,30,31]. On the contrary, HE specimens showed little t-m transformation. HE specimens showed significantly higher SBS values than SB or CT specimens (*p* < 0.05). These results are consistent with the findings of Kang et al. [32]. With Maxcem Elite, HE specimens showed a markedly (810%) higher adhesive strength than CT specimens, as well as a 105% higher adhesive strength than SB specimens. With MAZIC Cem, HE specimens showed a 90% higher adhesive strength than CT specimens, and a 40% higher adhesive strength than SB specimens. With RelyX U200, HE specimens showed a 120% higher adhesive strength than CT specimens, and a 25% higher adhesive strength than SB specimens. MAZIC Cem is an MDP-containing resin cement, and RelyX U200 is a phosphonic acid-based cement. Reports have shown that sandblasting or tribochemical coating, followed by the application of MDP-containing primers/resin cements, resulted in increased bonding strength [18,33]. Therefore, many commercial products such as adhesives, primers, and resin cements have incorporated 10-MDP, seeking to improve the bonding to zirconia prostheses [34]. A phosphonic acid-based cement has also exhibited statistically comparable bond strength to MDP-containing resin cements [2]. However, other studies have confirmed that the active parts of 10-MDP react with the zirconia surface, but exhibiting a predisposition to instability with aging [35]. The main problem of 10-MDP is hydrolytic degradation [12]. Because Maxcem Elite is not an MDP-containing resin cement or a phosphonic acid-based cement, it showed the lowest SBS values in the CT specimens. However, after hot-etching, Maxcem Elite showed a markedly increased adhesive strength. Therefore, the null hypothesis of the present study was rejected.

The clinical success and longevity of ceramic restorations rely on a suitable bond between the ceramic and dental substrate. An adequate surface roughness is necessary for cement interlocking and enhanced bonding [2,36]. The mechanical surface treatment recommended for ceramics is sandblasting with aluminum oxide particles or glass beads to improve the mechanical retention of the materials. Sandblasting produces roughening and irregularities on the substrate surface, which promotes the micromechanical interlocking of the resin. This surface treatment increases the adhesive area for bonding, thereby allowing the resin cement to flow into the surface. Ceramic surfaces abraded with aluminum oxide particles have been suggested as the preferred surface treatment method for high-strength ceramic materials, such as alumina and zirconia ceramics [20,37]. Previous studies have shown that sandblasting with aluminium oxide particles increases or decreases the strength of zirconia [38,39]. In addition, sandblasting can be affected by various factors, such as the injection pressure and time, particle shape, and size of the aluminium oxide particles [38,40]. The use of sandblasting presents a problem in terms of long-term stability due to surface phase transformation (t-m). However, the high-concentration HF etching method did not cause a phase transformation on the zirconia surface in a previous study [41]. Similarly, in our study, HE specimens did not show monoclinic phase transformation of the surface-treated zirconia.

The chemical surface treatment recommended for ceramics is acid etching, which enhances the bond integrity between glass or glass-ceramic restorations and resin cement. HF acid etching is a well-accepted pretreatment regimen that produces irregular surface topography. HF etching forms a porous structure on the ceramic surface by reacting with the silica matrix of the ceramic. The boundary areas of silica in the ceramic matrix dissolve at a higher rate than those within the exposed grains [29]. However, zirconia is not etched by HF at room temperature because it is a non-silica-based ceramic.

In previous investigations of the effect of various temperatures and various HF concentrations, immersion of zirconia into 9.5% HF produced similar surface roughness at 80 °C for 1 min and at 25 °C for 1 h. The surface irregularities increased with increasing immersion time and etching solution temperature [26]. Sriamporn et al. reported that HF can etch dental zirconia ceramics, creating micro-morphological changes, but a t-m phase transformation was induced on the etched zirconia surface. They assumed that a t-m phase transformation was caused by low temperature degradation, because the HF etching was applied with prolonged exposure to humidity at low temperature. In addition, Lee et al. showed that, as the concentration of HF increased to 10%, 20%, and 30% and the treatment time increased from 5 min to 30 min, the wettability of the zirconia surface increased, and SBS with resin cement also tended to increase [42]. Cho et al. reported that the SBS between zirconia and resin cement treated with a high concentration of nitric acid–hydrofluoric acid compound at 25 °C for 2 h showed higher SBS values than those treated with air abrasion and tribochemical silica coating [27]. In their findings, MDP-containing resin cement showed notably lower bond strength after acid etching. They hypothesized that chemical etching of zirconia could have eliminated the hydroxyl group on its surface, and hence reduced the SBS of the MDP-containing resin cement. They suggested that applying the right cement is more crucial than surface conditioning. In a previous study using the airborne-particle abrasion and HE methods together, it was reported that the surface roughness of zirconia increased [43]. However, Iijima et al. reported that flaws and microcracks caused by sandblasting and acid etching may be responsible for the decrease in the flexural strength of zirconia. A recent study showed that acid etching treatment of the zirconia surface can improve the adhesion of resin cement in clinical applications [44]. Another recent study reported that laser irradiation of pre-sintered zirconia surfaces showed the highest SBS values of a resin cement, while sandblasting of the pre-sintered and sintered zirconia surfaces did the lowest SBS values [45].

However, using high concentrations of HF at high temperatures is a very dangerous method, as it involves exposure to HF vapor [29,46,47]. The 9% HF gel used in our study, which is generally used in dentistry for glass or glass ceramic etching, could be applied in a small amount to the desired area and is relatively safer than immersion in a high-concentration HF solution. The triple-locking structure of the safe shell we used also had the effect of suppressing the rapid release of HF vapor, as the neutralizing gel was applied to the top of the safety container. 

There were some limitations to the present study. The present study did not investigate long-term water storage and thermocycling which can affect the adhesion and durability of zirconia prostheses. Future studies should include clinical studies to confirm the effect of hot-etching with 9% HF, and a study on shear bonding strength before and after thermocycling is warranted.

## 5. Conclusions

Zirconia ceramics are not readily etched using low-concentration HF at room temperature. However, we showed that the zirconia surface could be safely and effectively hot-etched using 9% HF gel in a safe shell. SEM and SR analyses showed that hot-etching with 9% HF distinctly affected the surface treatment of zirconia ceramics, as compared to CT specimens. XRD analysis revealed a transformation to the monoclinic phase only in the SB specimen. SBS values were significantly higher with self-adhesive resin cements (Maxcem Elite, MAZIC Cem, RelyX U200) in the HE group than in the SB group (*p* < 0.05). Within the limitations of the present study, we concluded that zirconia prostheses can be bonded micromechanically with resin cement, without deterioration of properties due to t-m transformation, using chemical acid etching with the Zirconia Etchant Cloud System. The long-term stability of resin cement to zirconia prostheses should be further evaluated.

## Figures and Tables

**Table 1:** Resin cements used in this study.

Product	Manufacturer	Composition
Maxcem Elite	Kerr Corporation Brea, CA, USA	GPDM, Monomers, Nonhazardous inert mineral fillers, Ytterbium fluoride, Activators, Stabilizers, and Colorants
MAZIC Cem	Vericom Co., Ltd. Chuncheon, Korea	Barium silicate, Fluorinated barium silicate, Fumed silica, MDP, Bis-GMA, Dimethacrylate, Catalyst, Stabilizer, Pigments
RelyX U200	3M ESPE, Maplewood, MN, USA	Methacrylate monomers containing phosphoric acid group, Methacrylate monomers, Silanated fillers, Alkaline (basic) fillers, Initiator components, Stabilizers, Rheological additives, Pigments

**Table 2:** Surface roughness (Ra, µm) and peak-to-valley roughness (Rt, µm) of control, SB and HE (mean ± standard deviation).

Surface Treatment	Surface Roughness Values, Ra (μm)	Peak to Valley Roughness, Rt (μm)
Control (CT)	0.190 ± 0.01	2.40 ± 0.28
Sandblast (SB)	0.320 ± 0.01	4.21 ± 0.42
HF etching (HE)	0.230 ± 0.01	3.03 ± 0.69

**Table 3:** Result of shear bond strength between surface treatments and resin cements (*p* < 0.05).

Surface Treatment	N	Maxcem Elite		MAZIC Cem		RelayX U200	
		Mean	SD	Mean	SD	Mean	SD
Control (CT)	15	0.76	0.35	4.93	1.42	3.33	1.27
Al_2_O_3_ Sandblast (SB)	15	3.38	2.09	6.80	2.11	6.00	0.78
HF Etching (HE)	15	6.98	1.81	9.53	1.50	7.27	1.32

**Table 4:** Comparison statistics of shear bond strength between surface treatments and resin cements.

Resin Cement	Comparing Surface Treatment	95% Confidence Interval			
		Mean Difference	Lower Bound	Upper Bound	*p*-Value
Maxcem Elite	Control–SB	−2.61358	−4.0424	−1.1848	0.000
	Control–HE	−6.21811	−7.6469	−4.7893	0.000
	SB–HE	−3.60453	−5.0333	−2.1757	0.000
MAZIC Cem	Control–SB	−1.87301	−3.3842	−0.3618	0.012
	Control–HE	−4.59795	−6.1092	−3.0867	0.000
	SB–HE	−2.72494	−4.2362	−1.2137	0.000
RelayX U200	Control–SB	−2.67207	−3.6954	−1.6487	0.000
	Control–HE	−4.24693	−5.2703	−3.2236	0.000
	SB–HE	−1.57485	−2.5982	−0.5515	0.002

## Data Availability

Not applicable.
